# Stress Distribution and Tooth Displacement Analysis of Maxillary Molar Distalization by Different Designs of Jig in a Finite Element Study

**DOI:** 10.30476/dentjods.2024.100556.2230

**Published:** 2025-03-01

**Authors:** Hooman Zarif Najafi, Hamid Reza Pakshir, Faezeh Bahraini

**Affiliations:** 1 Orthodontic Research Center, Shiraz University of Medical Sciences, Shiraz, Iran

**Keywords:** Finite element analysis, Orthodontic Appliances, Fixed, Tooth Movement Techniques

## Abstract

**Statement of the Problem:**

Despite the prevalence of CLII malocclusion, still the best mechanotherapy for non-extraction treatment is not verified.

**Purpose:**

The aim of the present study was to evaluate the stress distribution and tooth displacement during maxillary molar distalization with the aid of two different constructions of jigs in three different lever arm heights.

**Materials and Method:**

In this finite element study, models were meticulously constructed to represent the maxillary arch teeth (excluding the third molar), periodontal ligament, alveolar bone, maxillary brackets, main archwire, molar bands, jigs, and mini screws. These models were imported into Ansys software for simulation and analysis. Two different jig configurations with three different lever arm height were created. A 150-gram force was applied to simulate tooth movement, facilitate observation, and analyze its effects on oral components.

**Results:**

In various experimental configurations involving tow jigs with differing lever arm heights, the central incisor exhibited displacement characterized by lingual and distal crown tipping, along with evidence of intrusion. Concurrently, the first molar displayed lingual and distal tipping, as well as extrusion, across six distinct modalities.

**Conclusion:**

In the main, posterior teeth showed distal and lingual tipping and extrusion and anterior teeth demonstrated intrusion mesial and lingual tipping in all models. It seems the differences were due to different lever arm heights. Two types of jig had no significant effect on stress distribution and tooth movement.

## Introduction

Class II malocclusion is one of the most prevalent types of malocclusion [ 1 ]. The treatment strategy, i.e., growth modification [ 2 ], camouflage, and orthognathic surgery [ 3 ], is based on the severity of the problem and the patient’s developmental stage. 

Patients with less severe dentofacial deformity have a lesser need for orthognathic surgery [ 4 ]. Since dental compensation does not entail the cost and invasiveness of orthognathic surgery, it is preferred by patients [ 5 ].

Patients with mild to moderate CL II malocclusion can be treated through extraction, non-extraction, or maxillary dentition distalization [ 6 ]. Recently non-extraction treatment has been more popular among patients [ 7 ].

Several appliances, such as a pendulum [ 8 ], distal jet [ 9 ], carrier distalizer [ 10 ], iPanda [ 11 ], headgear [ 12 ], and so on can achieve maxillary molar distalization. Patient cooperation and anchorage loss are examples of the problems of these methods [ 13 - 14 ]. With the help of a temporary anchorage device, molar distalization is feasible without unwanted movement of the anterior segment [ 15 ]. 

Several case reports have evaluated intraoral molar distalization with a mini screw-supported sliding jig [ 16 - 17 ], lever arm [ 18 ], EZ slider auxiliary [ 19 ], and sectional jig assembly [ 20 ]. Maxillary molar distalization alongside canting in the occlusal plane [ 17 , 19 ], intrusion, extrusion, and rotation of molars, premolars, and canines [ 18 - 19 , 21 ], distal, buccal, and palatal tipping of posterior teeth [ 18 , 21 - 23 ], and incisors retroclination [ 21 , 23 ] have been reported. In addition, bodily molar distalization by directing the force through the center of resistance (CR) is achievable [ 11 , 23 ]. 

Finite element analysis (FEA) has been used to evaluate the different biomechanical aspects of force in orthodontics [ 24 - 26 ]. FEA can stimulate the object’s responses to orthodontic forces by assessing stress distribution at the periodontal ligament–alveolar bone interface [ 27 ]. 

Ammoury *et al.* [ 24 ] and Ueno *et al.* [ 28 ] investigated the maxillary molar distalization via direct and indirect anchorages, trans-palatal arch, and mini screws via FEA, respectively [ 24 , 28 ]. Other FEA studies also evaluated other maxillary molar distalization modalities like zygomatic gear [ 29 ], asymmetric headgear [ 30 ], and iPanda [ 31 ].

To the best of our knowledge, no study has evaluated the effect of molar distalization on continuous archwire using a sliding jig inserted into the headgear tube of the molar’s band and compared it with a sliding jig not inserted into the headgear tube of molar’s band regarding the different heights of sliding jig’s lever arm. Regarding the ease of use of this technique and its popularity, knowledge about its potential effects, like tipping and rotation of molar and anterior teeth, and vertical movements, such as intrusion or extrusion of teeth and canting or flattening of the occlusal plane, is mandatory.

Therefore, this study evaluated the effect of the stress distribution and tooth displacement during maxillary molar distalization with the aid of two different constructions of jigs in three different lever arm heights.

## Materials and Method

This study employs finite element analysis to assess molar distalization, utilizing two distinct jig types.

### Construction of Finite Element Model

The study protocol received approval from the Ethical Committee of Shiraz University of Medical Sciences (IR.SUMS.DENTAL.REC.1401.083). A three-dimensional (3D) model was generated using cone-beam computed tomography (CBCT) data with a resolution of 0.1 mm/ pixel from a patient seeking head radiologic assessment. Informed consent was obtained from the patient for participation.

### Patient Enrollment Criteria

The study enrolled a patient meeting specific criteria: absence of ongoing orthodontic treatment, complete permanent dentition except for the third molar (24). Exclusion criteria included periodontal issues with alveolar bone loss, restored teeth (32), impaction (24), abnormal root or crown morphology, root resorption, or craniofacial syndrome.

### Construction of 3D Model

This study employed Materialise Interactive Medical Image Control System software (MIMICS) to construct the 3D model. The periodontal ligament (PDL) thickness was set at 0.25 mm, assuming linear elasticity based on prior research. Various components such as teeth, alveolar bone, mini screw, distalization jig [0.8-mm stainless steel (SS)], arch wires (0.0160.022 inch SS), and brackets (0.0180.025-inch slot) were created. Relationships between these elements were established using contact elements. Materials were considered homogeneous and isotropic based on literature references.

### Finite Element (FE) Framework

The MIMICS-generated FE framework comprised 1,213,424 elements and 1,850,320 nodes.

### Three-dimensional Coordinate System and Boundary Conditions

Coordinate systems for all 3D frameworks utilized X (buccolingual), Y (anteroposterior), and Z (occlusogingival) planes. Negative values for X, Y, and Z indicated buccal, mesial, and downward displacements, respectively. Displacements and rotations were measured in the maxillary arch for specific dental landmarks. Surface-to -surface interactions between adjacent teeth were used to create the contact interfaces. The contacts between the brackets and the wires were presumed to be frictionless.

### Force Application

A distalization force of 150 grams was applied from the mini screw to the jig's lever arm using a coil spring. Spring elements connected nodes of the archwire to teeth nodes for facilitating sliding. Contact between brackets and wires involved three-dimensional surface-to-surface sliding contacts with a friction coefficient of 0.1. The Ansys software 2019 was utilized for finite element simulation.

### Distalization Modalities

### First distalization modality

**Figure 1:**
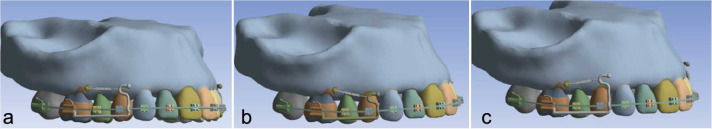
Jig type 1, **a:** Level lever arm, **b:** Short lever arm, **c:** Long lever arm

All the teeth were aligned. Brackets (3mm in width) [ 35 ] were applied on all teeth except the first and second molars, in which the molar’s band (4mm in width) [ 26 ] and tube (4mm in width) [ 26 ] were applied, respectively. Then 0.016*0.022 inch SS was applied. The mini screw (6mm in length and 1.5mm in diameter) was located between the second premolar and first molar [ 24 ] at the height of the first molar’s CR; it was used for direct anchorage distalization. A 0.9-mm round SS wire was used to construct the distalization jig [ 43 ]. Regarding the design, the distal extension of the jig was inserted and fitted into the headgear tube of molar band. The mesial extension had a helix that surrounded the main archwire, and the jig in the distal section had a lever arm (Figure 1).

The lever arm was set at different heights to explore the effects of various heights of the lever arm according to the mini screw and CR height. First, they were set at the same level to the miniscrew; second and third, the height of the jig’s lever arm was 2 mm higher and lower than the level of the mini screw, respectively.

### Second distalization modality

**Figure 2:**
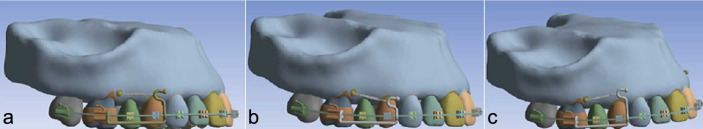
Jig type 2, **a:** Level lever arm, **b:** Short lever arm, **c:** Long lever arm

All the assumptions were similar to the first modality, except that it had no extension wire in the molar’s headgear tube, and the distal helix was considered a stop at the mesial of the headgear tube of the molar band (Figure 2).

## Results

**Figure 3:**
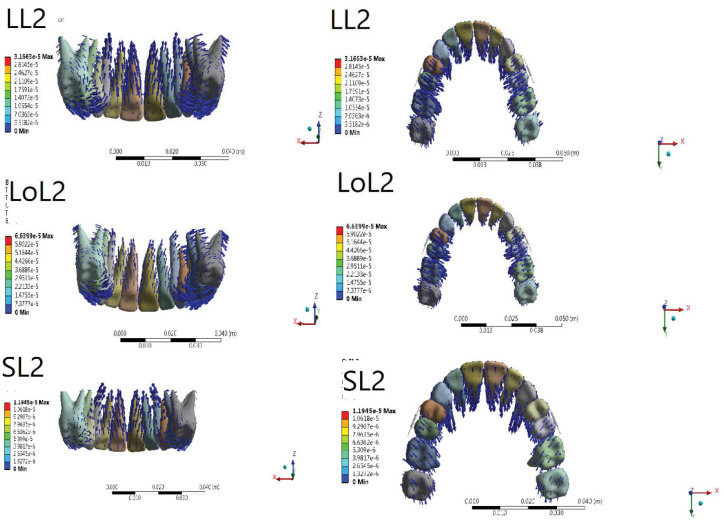
Displacement tendencies of the jig type 2 configuration in different views

**Figure 4:**
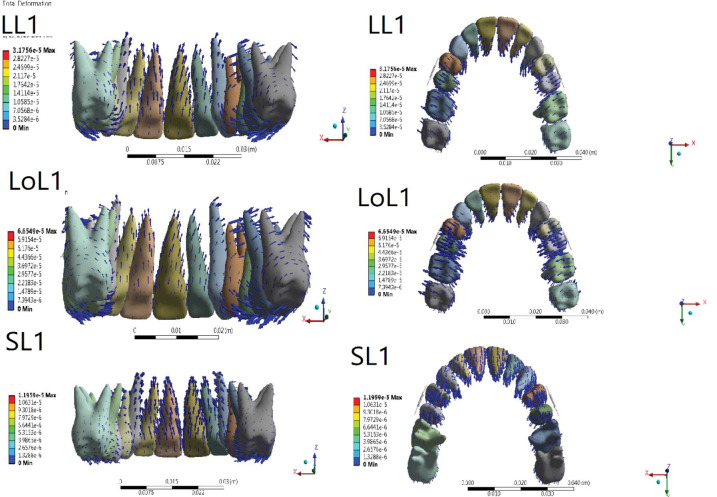
Displacement tendencies of the jig type 1 configuration in different views

Amount of tooth displacement in Y, X, and Z axis is demonstrated in Table 1. Figure 3 and 4 shows three-dimensional initial tooth displacements caused by the two types of jig at three different heights of the lever arm. 

**Table 1:** Young’s modulus and Poisson’s ratio of material properties

Component	Young’s modulus (MPa)*	Poisson’s ratio
Tooth [ 38 ]	20300	0.3
PDL [ 38 ]	0.667	0.49
Alveolar bone [ 38 ]	34000	0.26
Bracket/mini screw/wire/ Jig [ 24 ]	200000	0.3

* MPa: Mega Pascal

### Central Incisors Displacement

### Y-axis

Central incisors’ crown and root were displaced palatally with root displacement greater than the crown’s in all six modalities. Among different heights of the lever arm, the LL in both jig types showed the greatest amount of crown and root displacement (LL1: C= 3.9e-4, R= 9.8e-4, and LL2: C= 3.54 e-4, R= 9.3 e-4). The lowest amount of crown and root displacement belonged to the LoL (LoL1: C= 1.76 e-4, R= 8.8 e-4, and LoL2: C= 1.9 e-4, R = 8.5 e-4). In all types of lever arm height, crown and root displacement in type 1 jig were greater than in type 2 jig, except in LoL and SL where crown displacement in type 2 jig was greater (Figures 3-4, Table 2).

**Table 2:** Displacement of all teeth in X, Y, and Z directional axes

Tooth	LL1		LoL1		SL1		LL2		LoL2		SL2	
IE1	X	7.3e-5	X	9.3e-5	X	7.9 e-5	X	7.1e-5	X	1.1 e-4	X	7.7e-5
	Y	3.9e-4	Y	1.76 e-4	Y	1.95 e-4	Y	3.54 e-4	Y	1.9 e-4	Y	2.1 e-4
	Z	4.9e-4	Z	4.4 e-4	Z	5.76 e-4	Z	4.3 e-4	Z	4.1 e-4	X	5.55 e-4
R1	X	-2.6e-5	X	-6.9 e4	X	-1.1 e-4	X	-2.9 e-5	X	-7.2e-5	X	-1 e-4
	Y	9.8e-4	Y	8.8 e-4	Y	9.1 e-4	Y	9.3 e-4	Y	8.5 e-4	Y	8.6 e-4
	Z	1.2e-4	Z	9.8e-5	Z	3.8 e-5	Z	1.55 e-4	Z	1.1 e-4	Z	3.9e-5
CT3	X	1.3 e-4	X	3 e-4	X	-1.3 e-4	X	1.4 e-4	X	2.5 e-4	X	-1.3 e-4
	Y	2.1 e-4	Y	2.4 e-4	Y	3 e-4	Y	3.7 e-4	Y	2.8 e-4	Y	3 e-4
	Z	3.5 e-4	Z	3.5 e-4	Z	5.1 e-4	Z	3.9 e-4	Z	3.1 e-4	Z	4.9 e-4
R3	X	-5.6e-4	X	-3.4 e-4	X	2.1 e-4	X	-7.2 e-4	X	-3.7 e-4	X	2.1 e-4
	Y	8.2 e-4	Y	6.9 e-4	Y	8.8 e-4	Y	7.4 e-4	Y	6.1 e-4	Y	8.5 e-4
	Z	7.1e-5	Z	1.1 e-4	Z	4e-5	Z	7.2 e-4	Z	1.39 e-4	Z	4e-5
BC5	X	6.7 e-4	X	1e-3	X	3.22 e-4	X	7.2 e-4	X	1.15e-3	X	3 e-4
	Y	3.64 e-4	Y	5.4e-4	Y	2.6 e-4	Y	3.6 e-4	Y	5.1 e-4	Y	2.9 e-4
	Z	-6.2 e-4	Z	-6 e-4	Z	-3.4 e-4	Z	-6.2 e-4	Z	-9.2 e-4	Z	-3.5 e-4
R5	X	-8 e-4	X	-1.3e-3	X	-2.7 e-4	X	-7.5 e-4	X	-1.2e-3	X	-2.5 e-4
	Y	2.4 e-4	Y	-1.5 e-4	Y	6.1 e-4	Y	2.45 e-4	Y	-1.5 e-4	Y	6 e-4
	Z	-2.4 e-4	Z	-1.4 e-4	Z	-2.1 e-4	Z	-1.6 e-4	Z	-8.1e-5	Z	-2.1 e-4
MBC6	X	7 e-4	X	8.1e-4	X	3.4 e-4	X	6.8 e-4	X	9.8 e-4	X	3.2 e-4
	Y	1.6 e-4	Y	1.3 e-4	Y	2.75 e-4	Y	2.6 e-4	Y	2 e-4	Y	2.8 e-4
	Z	-5.8 e-4	Z	-6.5 e-4	Z	-3.9 e-4	Z	-5.6e-4	Z	-6.4 e-4	Z	-3.8 e-4
MBR6	X	-3.7 e-4	X	-5 e-4	X	-1.8 e-4	X	-3.4 e-4	X	-5 e-4	X	-1.5 e-4
	Y	-2.4 e-4	Y	-1.5 e-4	Y	-6.1e-5	Y	-9e-5	Y	-1.5 e-4	Y	-5e-5
	Z	-4.7 e-4	Z	-6.1 e-4	Z	-3.7 e-4	Z	-4.4 e-4	Z	-6.4 e-4	Z	-3.5 e-4
MBC7	X	3.3 e-4	X	3.7e-4	X	2.3 e-4	X	3.5 e-4	X	4.4 e-4	X	2.2 e-4
	Y	4.5 e-4	Y	1.8 e-4	Y	3 e-4	Y	2.5 e-4	Y	1.6 e-4	Y	3.2 e-4
	Z	-3.2 e-4	Z	-4.1 e-4	Z	-1.7e-4	Z	-3.1 e-4	Z	-3.4 e-4	Z	-1.6 e-4
MBR7	X	-4.3 e-4	X	-7.1 e-4	X	-2 e-4	X	-4 e-4	X	-6.7 e-4	X	-1.8 e-4
	Y	-1.5e-4	Y	-3.5 e-4	Y	-3.1 e-4	Y	-2.4 e-4	Y	-3.6 e-4	Y	-1.2 e-4
	Z	-3.2 e-4	Z	-4.5 e-4	Z	-2 e-4	Z	-3.3 e-4	Z	-4.7 e-4	Z	-1.7 e-4

X: + lingual, Y: + distal, Z: + apical, IE1: the central incisor edge, R1: the apex of central incisor, CT3: the canine cusp tip, R3: the canine apex, BC5: the second premolar cusp tip, R5: the second premolar apex, MBC6: the first molar mesiobuccal cusp tip, MBR6: the first molar mesiobuccal apex, MBC7: the second molar mesiobuccal cusp tip, MBR7: the second molar mesiobuccal apex

### X-axis

Central incisor’s root was displaced distally while the crown was displaced mesially in all six modalities. In type 1 jig, among different heights of the lever arm, the greatest amount of crown and root displacement belonged to LoL (C = 9.3e-5 and R = -1.15e-4), and the lowest amount of crown and root displacement belonged to the LL1 (C = 7.3e-5, R = -2.6e-5). In type 2 jig, the greatest amount of crown displacement occurred in LoL (1.1 e-4), and the greatest amount of root displacement was shown in SL (-1 e-4). Moreover, the lowest amount of crown and root displacement belonged to the LL (C= 7.1e-5, R= -2.9e-5). In LoL, crown and root displacement in type 2 jig was greater than in type 1 jig, but in SL, crown and root displacement in type 1 jig were greater than in type 2 jig. In LL, crown movement in type 1 jig was more than in type 2 jig, but root movement in type 2 jig was more than in type 1 jig (Figures 3-4, Table 2).

### Z-axis

Crown and root intrusion were observed in all six modalities. In both jig types, the greatest and lowest amounts of crown intrusion were recorded in SL and LoL, respectively (SL1= 5.76 e-4, SL2 = 5.55 e-4, LoL1 = 4.4 e-4, LoL2 = 4.1 e-4). In both jig types, the greatest and lowest amount of root intrusion was observed in LL and SL, respectively (LL1= 1.2 e-4, LL2= 1.55 e-4, and SL1= 3.8 e-5, SL2= 3.9 e-5). More crown and root intrusion was observed in type 1 jig compared with type 2 jig in all lever arm heights (Figures 3-4, Table 2).

### Canine Displacement

### Y-axis

**Figure 5:**
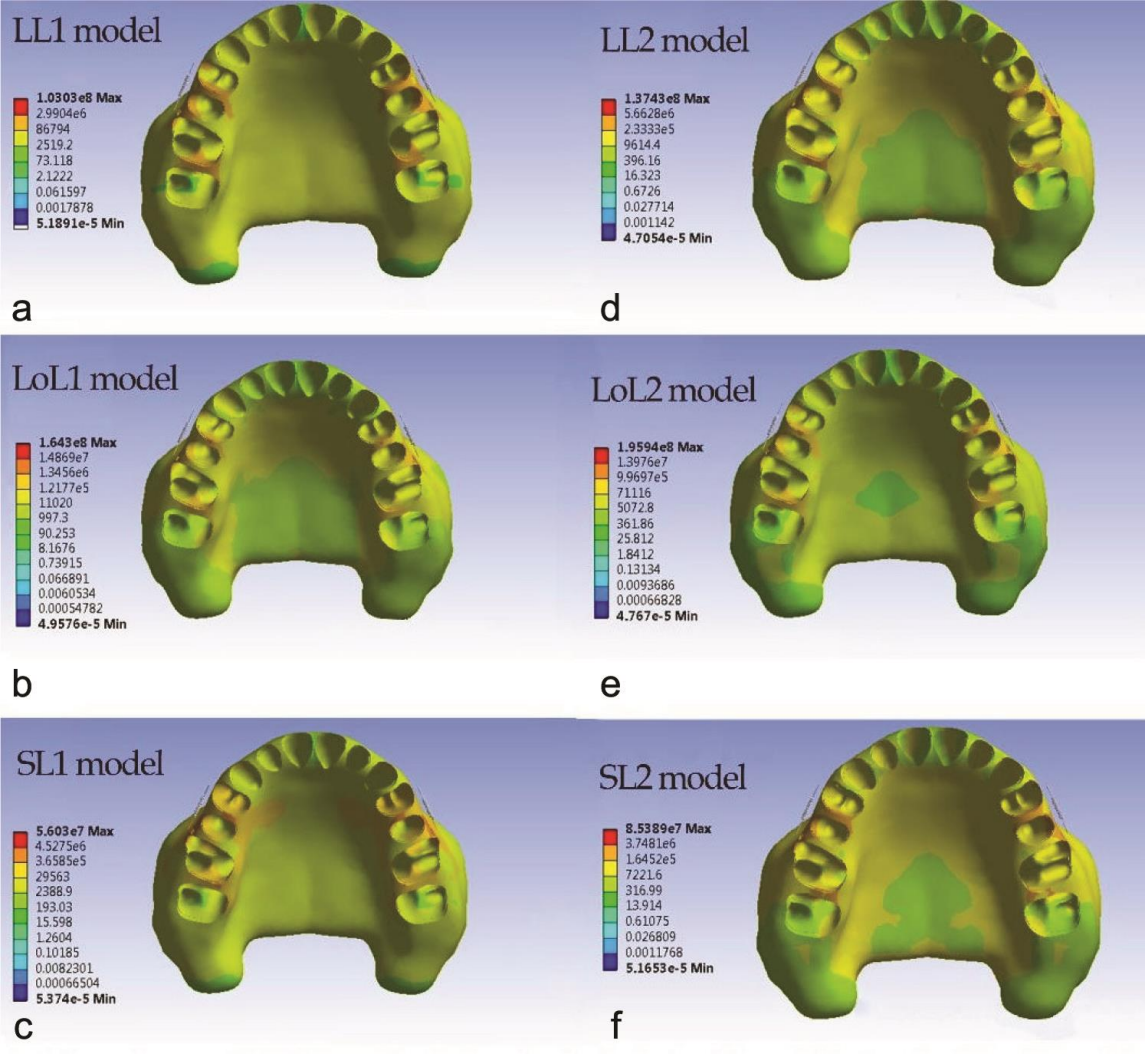
Von misses stress distribution within the maxillary bone in six modalities

**Figure 6:**
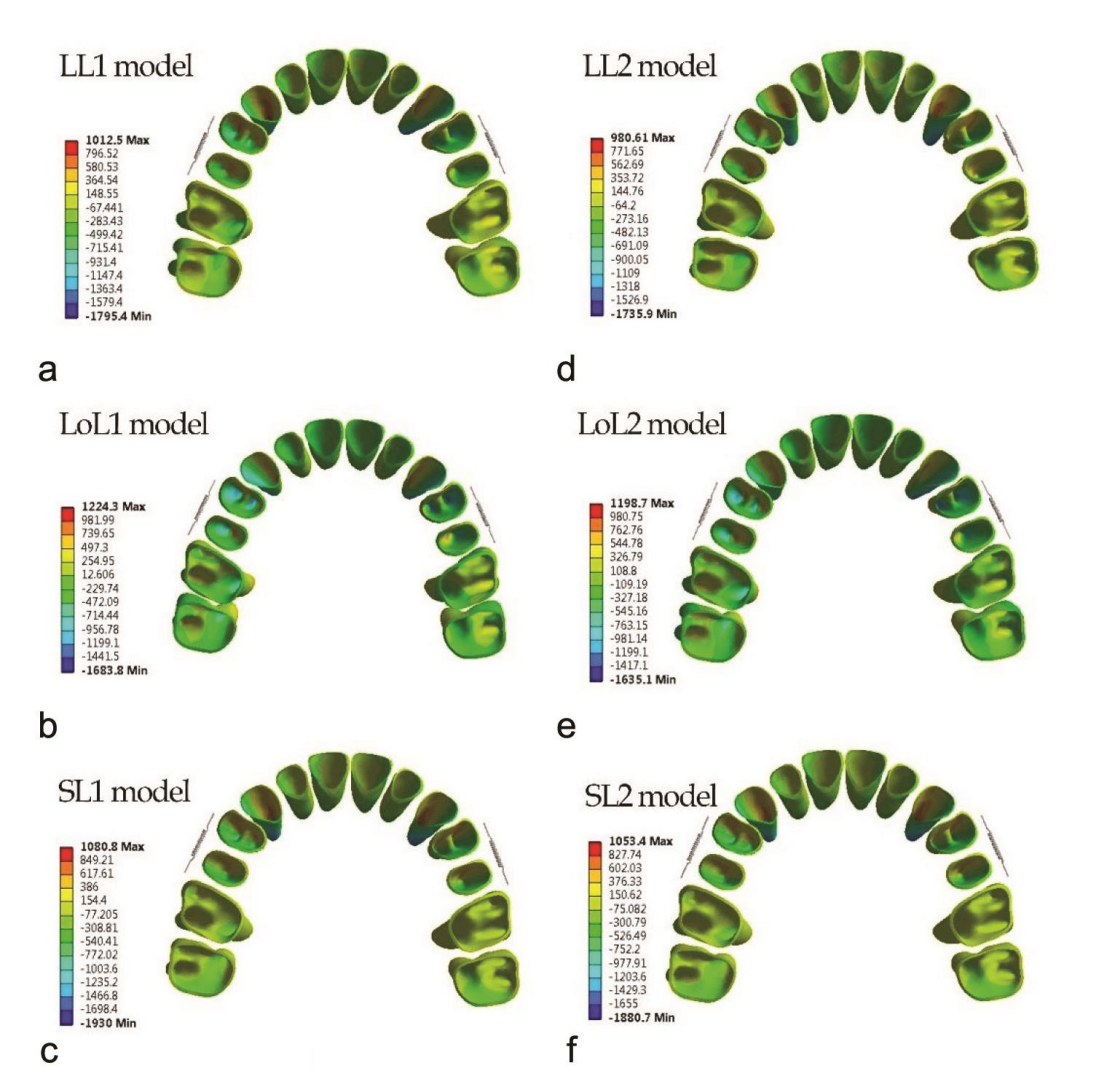
Maximum principal stress distribution within the periodontal ligament in six modalities

**Figure 7:**
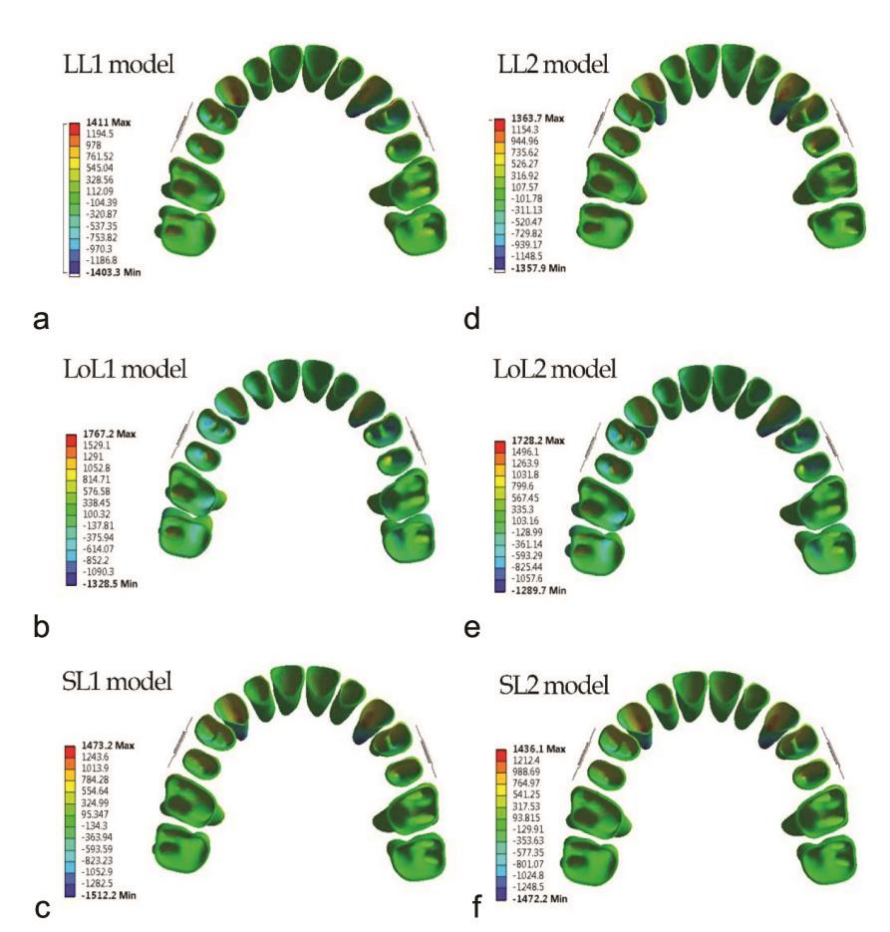
Minimum principal stress distribution within the periodontal ligament in six modalities

Canine’s crown and root were displaced distally with the root displacement greater than crown displacement in all six modalities. In type 1 jig, among different heights of the lever arm, the greatest amount of crown displacement belonged to SL (3e-4), and the greatest amount of root displacement belonged to SL (8.8 e-4). In this jig type, the lowest amount of crown and root displacement belonged to the LoL (C= 2.4e-4 and R= 6.9e-4). In type 2 jig, the greatest amount of crown displacement was shown in LL (3.7 e-4), and the greatest amount of root displacement was recorded in LL (7.4 e-4). In this jig type, the lowest amount of crown and root displacement belonged to the SL (C= 7.1e-5, R= 2.9e-5). In all lever arm heights, crown and root displacement in type 1 jig were greater than in type 2 jig, except in SL, where root displacement was equal in both jig types (Figures 3-7, Table 2).

### X-axis

Canine’s crown was displaced palatally, and the root was displaced buccally in both jig types in LL and LoL, but in SL, the canine’s crown was displaced buccally (SL1= -1.3 e-4, SL2= -2.5 e-4 mm), and the root was displaced palatally (SL1= 2.1 e-4, SL2= 3.7 e-4) in both jig types. The greatest amount of palatal crown displacement was shown in LoL (LoL1= 3 e-4 and LoL2 = 2.5 e-4), and the greatest amount of buccal root displacement was recorded in LL for both jig types (LL1= 5.6 e-4 and LL2= -7.2 e-4). In LoL and SL, crown and root displacement for type 2 jig were greater than in type 1 jig. In LL, crown movement in type 1 jig was more than type 2 jig, but root movement in type 2 jig was more than type 1 jig (Figures 3-4, Table 2).

### Z-axis

Crown and root intrusion were observed in all six modalities. In both jig types, the greatest and lowest crown intrusion was shown in SL and LoL, respectively (SL1= 5.1 e-4, SL2= 4.9 e-4, and LoL1= 3.5 e-4, LoL2= 3.1 e-4). More crown intrusion was observed in type 1 jig compared with type 2 jig. In both jig types, the greatest and lowest amount of root intrusion was shown in LoL and SL, respectively (LoL1= 1.1 e-4, LoL2= 1.39 e-4, and SL1= 4 e-5, SL2= 4 e-5. More root intrusion was observed in LL and LoL in type 2 jig compared with type 1 jig. Root intrusion in SL was the same for both jig types (Figures 3-4, Table 2).

### Second Premolar Displacement

### Y-axis

The second premolar’s crown and root were displaced distally in LL and SL for both jig types. The crown was displaced more distally in LL than the root, and vice versa in SL, where root displacement was more than the crown. However, in LoL, the crown was displaced distally while the root was displaced mesially. LoL showed the greatest amount of distal crown displacement for both jig types (LoL1= 5.4 e-4 and LoL2= 5.1 e-4), and SL showed the lowest amount of crown movement (SL1= 2.6 e-4, SL2= 2.9 e-4). In addition, SL showed the greatest amount of distal root displacement for both jig types (SL1= 6.1 e-4, SL2= 6e-4). LoL showed mesial root displacement for both jig types (LoL1= -1.5 e-4, LoL2= -1.5 e-4). In LL and SL, the amount of crown and root displacement was greater for jig type 2 compared with jig type 1, and in LoL, crown, and root displacement were almost equal for both jig types (Figures 3-4, Table 2).

### X-axis

The root displacement was greater than the crown displacement for both jig types in LL and LoL. In contrast, SL showed a greater amount of crown displacement compared with root displacement. For both jig types, the greatest amount of palatal crown movement and buccal root movement was observed in LoL (LoL1: C= 1e-3, R= -1.3e-3 and LoL2: C= 1.15e-3, R= -1.2e-3). For both jig types, the lowest amount of palatal crown and buccal root movements were observed in SL (SL1: C= 3.22e-4, R= -2.7e-4 and SL2: C= 3 e-4, R= -2.5e-4). In LL and LoL, crown displacement was greater for type 2 jig compared with type 1 jig; however, in SL, crown displacement in type 1 jig was greater than in type 2 jig. In all lever arm heights, root displacement in type 1 jig was greater than in type 2 jig (Figures 3-4, Table 2).

### Z-axis

Crown and root extrusion was observed in all six modalities. In each jig type, the greatest and lowest amount of crown extrusion was shown in LoL and SL (LoL1= -6.2 e-4, LoL2= -9.2 e-4, and SL1= 2.1 e-4, SL2 = -3.5 e-4), respectively. In LL, more extrusion was observed in type 1 jig compared with type 2 jig. At other lever arm heights, extrusion was greater in type 2 jig. In each jig type, the greatest and lowest root extrusion was observed in SL1= -2.1 e-4 and SL2= -2.1 e-4, and LoL1= -1.4e-4 and LoL2= -8.1 e-5), respectively. In LL and SL, root extrusion was the same in both jig types, and in LoL, root extrusion was greater in type 1 jig (Figures 3-4, Table 2).

### First Molar Displacement

### Y-axis

The first molar’s crown was displaced distally, while the roots were displaced mesially in all six modalities. For both jig types, the greatest and lowest amounts of distal crown displacement was observed in SL and LoL, respectively (SL1= 2.75e-4, SL2= 2.8e-4 and LoL1= 1.3e-4, LoL2= 2 e-4), considering mesiobuccal cusp. For type 1 jig, SL showed the greatest, and SL showed the lowest amount of mesial root displacement considering mesiobuccal root (-6.1e-4 and -1e-4, respectively). For type 2 jig, LL showed the greatest, and LoL showed the lowest amount of mesial root displacement (-9e-4 and -1.5 e-4, respectively). In LL, crown displacement was greater in type 2 jig compared with type 1 jig, but root displacement was greater in type 1 jig. The amounts of crown and root displacement in type 1 and 2 jigs were almost equal in SL and LoL. The crown displacement in LoL was greater in type 2 jig compared with type 1 jig, but in SL, the root displacement was greater in type 1 jig compared with type 2 jig (Figures 3-4, Table 2).

### X-axis

The first molar’s crown was displaced palatally, while the roots were displaced buccally in all six modalities. For both jig types, the greatest amount of palatal crown displacement was observed in LoL (LoL1= 8.1 e-4 and LoL2= 9.8 e-4), and the lowest amount of palatal crown displacement was recorded in SL (SL1= 3.4 e-4 and SL2= 3.2 e-4), considering mesiobuccal cusp. For both jig types, the greatest amount of mesiobuccal root displacement was shown in LoL (LoL1= -5 e-4, LoL2= -5 e-4), and the lowest amount of mesiobuccal root displacement was observed in SL (SL1= -1.8 e-4, SL2= -1.5 e-4). At all lever arm heights, crown and root displacement in type 1 jig was greater than in type 2 jig, except in LL and LoL, where root displacement was equal in both jig types (Figures 3-4, Table 2).

### The von Mises stress

The alveolar bone stress distribution pattern was similar in both jig types, but in type 2 jig, it was greater than in type 1 jig (Figure 5). Some differences were found between different lever arm heights.

In LL, the most stress concentration was observed in the middle third of proximal bone between the first and second premolars. The next most stress concentrations were observed in interproximal palatal two-thirds of alveolar bone between the first and second premolars type 1 jig, the interproximal palatal alveolar bone between the first and second premolars in type 2 jig, the cervical third of the buccal alveolar bone of the second premolar, and the mesiobuccal third of alveolar bone of the first molar. The palatal half of the interproximal alveolar bone between the first and second molars exhibited similar stress concentrations.

In LoL, the most stress concentrations were observed in the palatal half of the interproximal alveolar bone between the first and second molars, between premolars, and the buccal alveolar bone crest of the second premolar, and the mesiobuccal alveolar bone crest of the first molar (Figure 5). 

In SL, the most stress concentration was observed in the middle third of interproximal space between premolars. The second most stress concentrations were observed in the alveolar bone crest third of the second premolar, the mesiobuccal alveolar bone crest of the first premolar and first molar, and interproximal space between premolars and molars.

The lowest von Mises stress was located in the inter-proximal bone between central incisors in all modalities (Figure 5).

### Principal Stress

The general pattern of maximum and minimum principal stress in type 1 and 2 jigs was similar, but some differences were observed among lever arm heights (Figures 6-7). 

In LL in both jig modalities, a higher amount of tensile stress was found in the mesial half of the canine and the first and second premolars’ PDL; in premolars, the apical half of the mesial aspect of PDL stress had the highest stress. The cervical half of the mesial aspect of PDL stress had the highest stress in canines. Incisors showed the lowest level of tensile stress. Molars had greater tensile stress on the palatal side of buccal roots. The compressive stress was greatest in the distal aspect of canine PDL, especially in the apical third of PDL. Compressive stress decreased from the first premolar to the second molar. Compressive stress was greater in the mesial half of the PDL for premolars, and for molars, it was greater in the buccal aspect of PDL for all roots. The compressive stress distribution pattern in LoL and SL was repeated.

In the LoL modality, in both jigs, the greatest amount of tensile stress was in the apical third of the first premolar in the mesial aspect of PDL. Moreover, for other teeth, the stress distribution was as follows in descending order: the mesial aspect of canine PDL, the apical third of the mesial aspect of the second premolar, and the buccopalatal aspect of molar PDL. The compressive stress pattern of distribution was like the LL modality.

In the SL modality in both jigs, the greatest amount of tensile stress was in the apical third of canines in the mesial aspect of PDL. For other teeth, the stress distribution in descending order was as follows: the mesial aspect of the second premolar PDL, the apical third of the mesial aspect of the first premolar, and the buccopalatal aspect of the molar PDL. The minimum principal stress was found in central incisors. The minimum principal stress in LL was greater than or equal to SL, and LoL had the least minimum and maximum principal stress in the PDL of all teeth. Maximum principal stress in SL was greater than or equal to LL (Figures 6-7). 

## Discussion

In the present study, the 3D reconstruction of molar distalization using a mini screw and two jig types with different lever arm heights was simulated with the aid of FEA. The study provides insight into the biomechanical effects during distalization. This study further investigated the better approach in CL II malocclusion camouflage treatment. 

Several studies evaluated various methods of non-compliance distalization with or without using a mini screw [ 44 - 45 ]. Adverse effects such as anchorage loss, which manifest as a protrusion or increased overjet [ 44 - 45 ], distal molar tipping [ 45 ], and extrusion [ 44 ] are examples of shortcomings of the molar distalization without a mini screw. Using a mini screw solved some shortcomings, such as anchorage loss, but the molar tipping and extrusion are still unresolved [ 18 - 19 , 46 ]. Up to now, to the best of our knowledge, no study has evaluated the effect of a modified jig with different lever arm heights on dentitions, so the purpose of the present research was to evaluate tooth displacement and stress distribution of maxillary dentition and alveolar bone using different jig configurations and lever arm heights. 

Our findings from FEA revealed that during molar distalization, there was concurrent distal and buccal tipping of molars. Additionally, distalization of premolars and canines resulted in distal tipping, extrusion, and lingual tipping, and lingual tipping with intrusion, respectively and incisors showed lingual tipping and intrusion. Based on the results regarding the jig configuration and lever arm height, the greatest differences were observed among different lever arm heights, and the jig configuration had little effect on the results. This study showed molar distalization via uncontrolled distal crown tipping in all six modalities (two jig configurations and different lever arm heights), consistent with previous studies regarding molar distal tipping [ 47 - 48 ]. In addition, lingual tipping and extrusion of the first molar crown were observed in all modalities in the present study. Similarly, Yu *et al.* [ 39 ], in a FEA study, evaluated different modalities for molar distalization. They displayed uncontrolled distal tipping and extrusion during molar distalization in a modality, which applied distalization force to the hook between the canine and lateral incisor to the buccal mini screw between the second premolar and first molar [ 39 ]. The results of type 1 jig wire were contrary to our hypothesis, which postulated that engaging a stiff and almost full-dimension SS as a jig in the molar’s headgear tube could create a counterclockwise moment by engaging the wire at the edges of the headgear tube to prevent uncontrolled distal tipping and may lead to molar bodily movement. However, in both jig types, in LL, the force line of action was expected to pass through the first molar CR, leading to the first molar’s bodily displacement. In type 2 jig in SL and LoL, a concentric force of the line of action was expected.

Applying the force from the lever arm with different heights to the mini screw could pull the lever arm so that the proximal extension of the jig would be pulled toward the apical direction, and the distal extension of the jig would be pulled occlusally. Therefore, in the distal extension, an extrusive force was applied to the teeth, which could cause lingual crown tipping to the tooth CR in the buccal aspect. 

An uncontrolled clinical study showed that molar distal crown tipping and extrusion occurred by Applying a distal force to the lever arm, positioned at the same level as the buccally located mini screw, situated between the maxillary second premolar and the first molar, consistent with the present study [ 49 ]. Compared with the current research, two FEA research showed intrusion and buccal tipping displacement for molars [ 31 , 50 ]. In one of them, distalization force with a different angle relative to the occlusal plane was used to distalize maxillary teeth; the force was applied from the archwire between the canine and first premolar to the buccal mini screw located apically between the mandibular second premolar and the first, and in other, iPand and a mini screw were used in the depth of the palatal vault. In each case, the force exerted exhibited an intrusive vector. Contrary to our study's findings, a separate investigation comparing molar distalization via K-loop and buccal mini screw revealed lesser degrees of distal tipping, greater bodily displacement, extrusion in the mesial cusps, and intrusion in the distal cusps. The researchers claimed that the K loop caused good control of the moment-to-force ratio, leading to the control of tooth movements [ 51 ].

According to the findings of the current study, central incisor displacement was controlled by crown lingual tipping and intrusion in all modalities, in which a greater amount of lingual displacement was observed in LL for both jig configurations. The greater amount of lingual tipping could be because the force vector was horizontal, and there was no force division in the vertical vector. The greater amount of intrusion in SL might be attributed to a greater amount of apical displacement of the archwire. This finding is consistent with the result of an FEA study by Yu *et al.* [ 39 ], in the modality that the level of the buccal mini screw was higher than the hook level [ 39 ]. In addition, Kawamura *et al.* [ 52 ] noted lingual displacement and extrusion of incisors, which differed from our study. They highlighted that the highest extrusion was observed when the force angle relative to the occlusal plane was greatest (30°). This was attributed to clearance gap and elastic wire deflection, factors not considered in our study [ 52 ]. Hedayati *et al.* [ 53 ] found that extrusion or intrusion of incisors could be varied because of differences in the mini screw and lever arm height [ 53 ].

In our study, the canine was displaced via controlled distal tipping, intrusion, and uncontrolled lingual tipping. In the LL modality, uncontrolled buccal crown tipping has occurred. Like the central incisor, the greater amount of intrusion and displacement in the Y axis belonged to SL, and it could happen for the same reason as the incisors. In line with our study, a clinical study found canine distalization and distal tipping; the carrier motion appliance was used, and Cl II elastic was applied from the maxillary canine and mandibular first molar [ 54 ]. In an FEA study, distal and palatal displacements of canine were noticed; however, the type of tooth movement, i.e., translation or tipping, was not specified [ 21 ]. Unlike the present study, no buccolingual and mesiodistal direction displacement and a slight amount of extrusion were reported in the K loop-assisted molar distalization analysis. The reason might be the good control of the moment-to-force ratio, the equivalent force system, and the equilibrium force [ 51 ].

The second premolar was displaced distally through controlled tipping, except in the LoL modality, which showed uncontrolled distal tipping. The deflection of the wire caused by the traction of the lever arm tends to move the crown mesially and the root distally, resembling a confrontation of distal forces. This deflection could potentially account for this phenomenon. This deflection also caused extrusion of this tooth, which was observed in the SL and LoL at the lowest and greatest amounts of extrusion, respectively. The greatest amount of crown lingual tipping in LoL may be related to the greatest amount of extrusive force located buccally relative to the tooth CR. The minimum difference in root and crown movement in LL modality may be due to the low deflection in the archwire, which may cause bracket prescription to be expressed more effectively. Consistent with our study, Cambiano *et al.* [ 55 ] used a pendulum appliance with skeletal anchorage premolar extrusion and distal movement and concluded that this extrusion was due to the extrusion of the first molar, which was transmitted to the premolars through transseptal fibers [ 55 ]. Tekale *et al.* [ 51 ] evaluated the K loop for molar distalization, and their result indicated that overall intrusive and distal displacement of the second premolar was insignificant because the second premolar was not included in the force system. The K loop was located in the canine bracket and molar tube, and the premolars were not ligated [ 51 ]. 

In the current study, the maximum stress was observed in the interproximal bone between premolars (1.8778e7 Pascal) in LoL1, which was much lower than the ultimate tensile stress of the alveolar bone (135 Pascal) [ 56 ]. Accordingly, the stress was too low in all modalities to cause bone defects, suggesting that the risk of alveolar bone defect is low, concerning the force and biomechanics used in this study. Stress distribution in the alveolar bone was higher even in LoL than in SL and LL, indicating that LoL could be safer than the other two lever arm heights regarding alveolar bone. In all modalities, tensile and compressive stress distribution was consistent with tooth movement, indicating distal tipping, intrusion in the second premolar and molar, extrusion in the canine, and extrusion and lingual tipping in the incisor. In all six modalities, compressive stresses in the apex area of canine PDL were greater than other PDL areas in canine, consistent with canine intrusion. Moreover, compressive stress was lower in the apex area in the PDL of the second premolar and canine, indicating tooth extrusion. The greater amounts of tensile and compressive stresses in all teeth were observed in the mesial and distal aspects of PDL, respectively, suggesting tooth distal tipping. This finding is in contrast to the Sujaritwanid *et al.* [ 31 ] study. Using iPanda appliance and skeletal anchorage, they showed force distribution along the distal root surface of molar teeth and increased compression stress in the buccal crown of molars [ 31 ]. In the present study, LL force was in line with the first molar’s CR, but equal force distribute-on in the distal area of the molar’s PDL (translation) was not observed, which does not coincide with Worms *et al.* [ 57 ] study. They used a headgear with a different force line of action; when the force line of action was passed through molar’s trifurcation, translation occurred [ 57 ]. 

This study aimed to find the treatment modality with little unwanted consequences. In all treatment modalities, molar distal tipping and extrusion happened. Mohamed *et al.* [ 58 ] reviewed different molar distalization methods using skeletal anchorage, reporting molar distal tipping, intrusion, and extrusion based on the treatment modality [ 58 ]. In a systematic review, Soheilifar *et al.* [ 59 ] found little differences regarding molar distalization and tipping between conventional and skeletal anchorage [ 59 ]. 

A more effective approach involves recognizing the limitations of the method and making decisions based on the specific clinical situation at hand.

Since this was an FEA study, the results should be interpreted with caution. FEA has its own limitations and cannot express variations in the human anatomy that can affect the clinical results. The accuracy of the FEA studies is just like the assumptions that are applied. Further clinical studies are recommended. However, the results of the present study could help clinicians choose a relevant treatment among various treatment modalities based on the clinical situation.

## Conclusion

The current study disclosed biomechanical aspect of maxillary molar distalization using mini screw and jig. Based on the findings, in Z axis, jig type 1 had roughly greater amount of tooth displacement than jig type 2, also LoL and SL had greatest and lowest amount of tooth movement in posterior teeth, respectively. SL and LoL had greatest and lowest amount of tooth movement in anterior teeth, respectively. The impact of the jig type appeared to have a greater influence on the X and Y axes compared to the Z axis. The pattern of stress distribution was nearly the same for all six modalities studied.

### Abbreviations

FE: Finite element; FEA: Finite element analysis; FEA: Finite element method, LoL: Long lever arm, LL: Level lever arm, SL: Short lever arm, PDL: Periodontal ligament, CR: Center of resistance, CBCT: Cone-beam computed tomography, 3D: Three dimensional, MIMICS Materialise Interactive Medical Image Control System, SS: Stainless steel. 
